# A newly discovered radiation of endoparasitic gastropods and their coevolution with asteroid hosts in Antarctica

**DOI:** 10.1186/s12862-019-1499-8

**Published:** 2019-09-18

**Authors:** Kara K. S. Layton, Greg W. Rouse, Nerida G. Wilson

**Affiliations:** 1 Centre for Evolutionary Biology, The University of Western Australia, Crawley, WA 6007 Australia; 2 Molecular Systematics Unit, Western Australian Museum, Welshpool DC, WA 6986 Australia; 3 Present Address: Department of Ocean Sciences, Memorial University of Newfoundland, St. John’s, NF A1C 5S7 Canada; 4 Scripps Institution of Oceanography, University of California San Diego, La Jolla, CA 92093 USA

**Keywords:** *Asterophila*, Endoparasites, Coevolution, Asteroidea, Antarctica

## Abstract

**Background:**

Marine invertebrates are abundant and diverse on the continental shelf in Antarctica, but little is known about their parasitic counterparts. Endoparasites are especially understudied because they often possess highly modified body plans that pose problems for their identification. *Asterophila*, a genus of endoparasitic gastropod in the family Eulimidae, forms cysts in the arms and central discs of asteroid sea stars. There are currently four known species in this genus, one of which has been described from the Antarctic Peninsula (*A. perknasteri*). This study employs molecular and morphological data to investigate the diversity of *Asterophila* in Antarctica and explore cophylogenetic patterns between host and parasite.

**Results:**

A maximum-likelihood phylogeny of *Asterophila* and subsequent species-delimitation analysis uncovered nine well-supported putative species, eight of which are new to science. Most *Asterophila* species were found on a single host species, but four species were found on multiple hosts from one or two closely related genera, showing phylogenetic conservatism of host use. Both distance-based and event-based cophylogenetic analyses uncovered a strong signal of coevolution in this system, but most associations were explained by non-cospeciation events.

**Discussion:**

The prevalence of duplication and host-switching events in *Asterophila* and its asteroid hosts suggests that synchronous evolution may be rare even in obligate endoparasitic systems. The apparent restricted distribution of *Asterophila* from around the Scotia Arc may be an artefact of concentrated sampling in the area and a low obvious prevalence of infection. Given the richness of parasites on a global scale, their role in promoting host diversification, and the threat of their loss through coextinction, future work should continue to investigate parasite diversity and coevolution in vulnerable ecosystems.

**Electronic supplementary material:**

The online version of this article (10.1186/s12862-019-1499-8) contains supplementary material, which is available to authorized users.

## Background

Antarctica is a unique geographic region that experiences extreme environmental conditions and ongoing threats from a changing climate, with some of the fastest warming waters globally (e.g. [1, 2]). Historically, repeated glacial cycles and fragmentation of populations in the region have promoted speciation in many groups (e.g. [3, 4]) and as a result Antarctica and the surrounding sub-Antarctic islands boast a rich diversity of marine life (e.g. [5, 6]). In fact, recent work has identified the Antarctic Peninsula and sub-Antarctic islands as centres of marine diversity for notothenoid fishes [7] as well as marine invertebrates, including pycnogonids [8] and sponges [9]. Some benthic marine invertebrate groups are more well-represented than others in Antarctica, including echinoderms, which are abundant and diverse on the continental shelf (e.g. [10, 11]). Echinoderms are also important as hosts for parasites and commensal organisms, but although these echinoderm-parasite interactions have been studied in temperate and tropical systems, they have been rarely documented and studied in Antarctica (but see [12, 13]).

*Asterophila* [14], a genus of eulimid gastropod, is exclusively endoparasitic in asteroid sea stars. There are currently three described members in this genus, *A. japonica* [14] from the NorthWest Pacific, *A. perknasteri* [15] from Antarctica, *A. rathbunasteri* [15] from California, as well as a fourth undescribed species from the Kermadec Trench [15]. The level of host specificity varies between congenerics, but may reflect a lack of knowledge of host associations in this genus. For instance, *A. japonica* has been found in eight host species from four families and three orders [16] while *A. perknasteri* has been found from three different *Perknaster* species [15] and *A. rathbunasteri* has been found only from a single host (*Rathbunaster californicus*) [15]. Because these parasites display a highly simplified body plan for their endoparasitic lifestyle, only a few morphological characters have been shown to be informative between species [16]. Additionally, no prior study has employed molecular data for exploring phylogenetic relationships in this genus, thus much remains unknown. In fact, little is known about Antarctic eulimids in general, despite a potentially high diversity of species in the region [12].

Parasites comprise a large portion of global biodiversity and they may be important in driving allopatric diversification in their hosts [17, 18]. Additionally, when speciation between host and parasite is tightly coupled, a loss of the host lineage may result in a loss of associated species (co-extinction) [19], thereby having significant impacts on total biodiversity loss [20]. The close association between endoparasitic *Asterophila* and asteroid hosts lends itself to exploring co-phylogenetic patterns and determining whether these groups are evolving in synchrony and which coevolutionary events are promoting diversification in this system. Early studies of coevolutionary biology found evidence for strict cospeciation between host and parasite (e.g. gophers and lice [21]) and it is thought that cospeciation plays a particularly important role in endoparasitic taxa. However, recent work has challenged this idea by showing that host-switching and duplication events are more prevalent than cospeciation in other host-parasite systems (e.g. myzostomid worms and crinoids [22], nematodes and stick insects [23]), and some endoparasites show host promiscuity (e.g. copepods and nudibranchs [24]). Although echinoderm-parasitizing eulimid gastropods are diverse and exhibit a variety of parasitic lifestyles [25, 26], most prior work has focused only on the morphology and ecology of this clade and little is known about the evolution of parasitism in the group. Moreover, recent work has shown that parasitism and symbiosis are more common in Antarctica than once thought (e.g. [12, 13]).

Recent sampling in Antarctica uncovered a range of seastar hosts from different taxonomic groups that were parasitized by *Asterophila,* prompting us to explore diversity in this genus and investigate cophylogenetic patterns between parasites and hosts. This study employs molecular and morphological data for phylogenetic analysis and species delimitation. These phylogenetic methods shed light on an apparent radiation of *Asterophila* in Antarctica and aid in determining whether speciation in asteroid hosts has been paralleled by *Asterophila*. This work extends our knowledge of diversity in *Asterophila* and provides insight into the coevolutionary events promoting diversification in this system.

## Results

### *Asterophila* occurrence and distribution

**Fig. 1:**
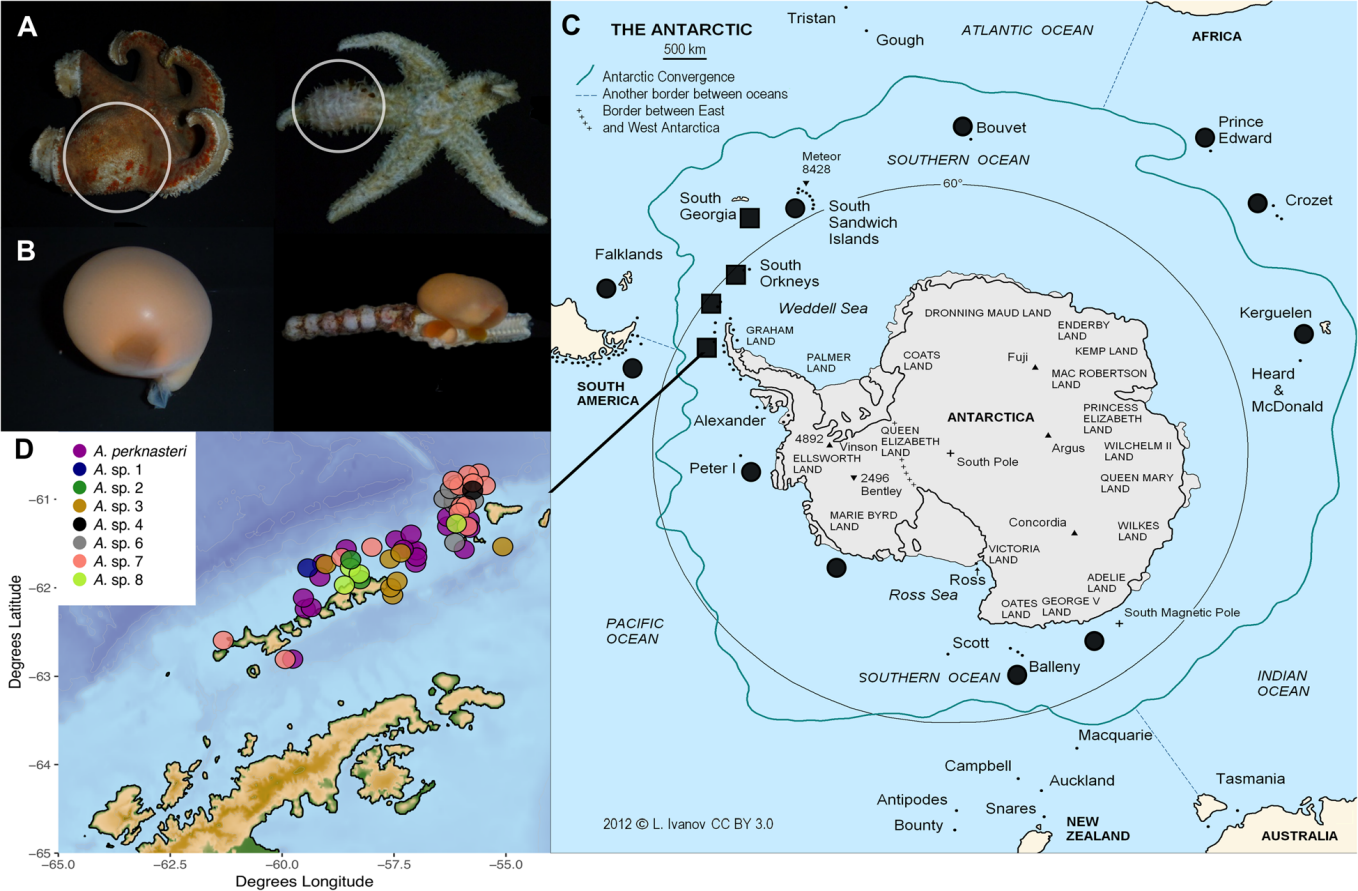
*Asterophila* distribution in Antarctica. **a** Host specimens infected by *Asterophila* (left: P00407, right: P00340)- white circles indicate cysts on the asteroid arm. **b** *Asterophila* specimens retrieved from hosts (left: M13618, right: M12882). **c** Localities marked with a square denote locations where *Asterophila* was present, and localities marked with a circle represent locations where hosts were sampled but *Asterophila* was absent (Wikimedia Commons contributors). **d** Detailed sampling map showing co-distribution of *Asterophila* species at South Shetland Islands created with the R package *ggplot2* [27] and with bathymetry data from the National Oceanic and Atmospheric Administration (NOAA) in the R package *marmap* [28]

A total of 61 *Asterophila* parasites were recovered from 38 hosts forming large and noticeable cysts on the aboral side of the host in the arms or near the central disc. *Asterophila* was found between the epidermis and coelomic lining, with no affiliation to specific organs or body systems. This description is congruent with Sasaki et al. [16] who describe the anatomy of *Asterophila japonica* in detail. Roughly 1500 potential hosts were collected in trawls on each cruise, and therefore an occurrence of only 38 infected hosts suggests the prevalence of parasitism in this group is low, though it may have been that only obvious infections were noted. Infected hosts and parasites were only recovered from near the Antarctic Peninsula, at Shetland Islands, Elephant Island, Shag Rocks and South Georgia (Fig. 1), despite sampling at various locations around the continent. An additional host with parasite (BIC-SIO E5030) was discovered and imaged from South Orkney in 2011, but the specimen was not included in these analyses.

### Phylogenetic reconstruction and species delimitation

**Fig. 2:**
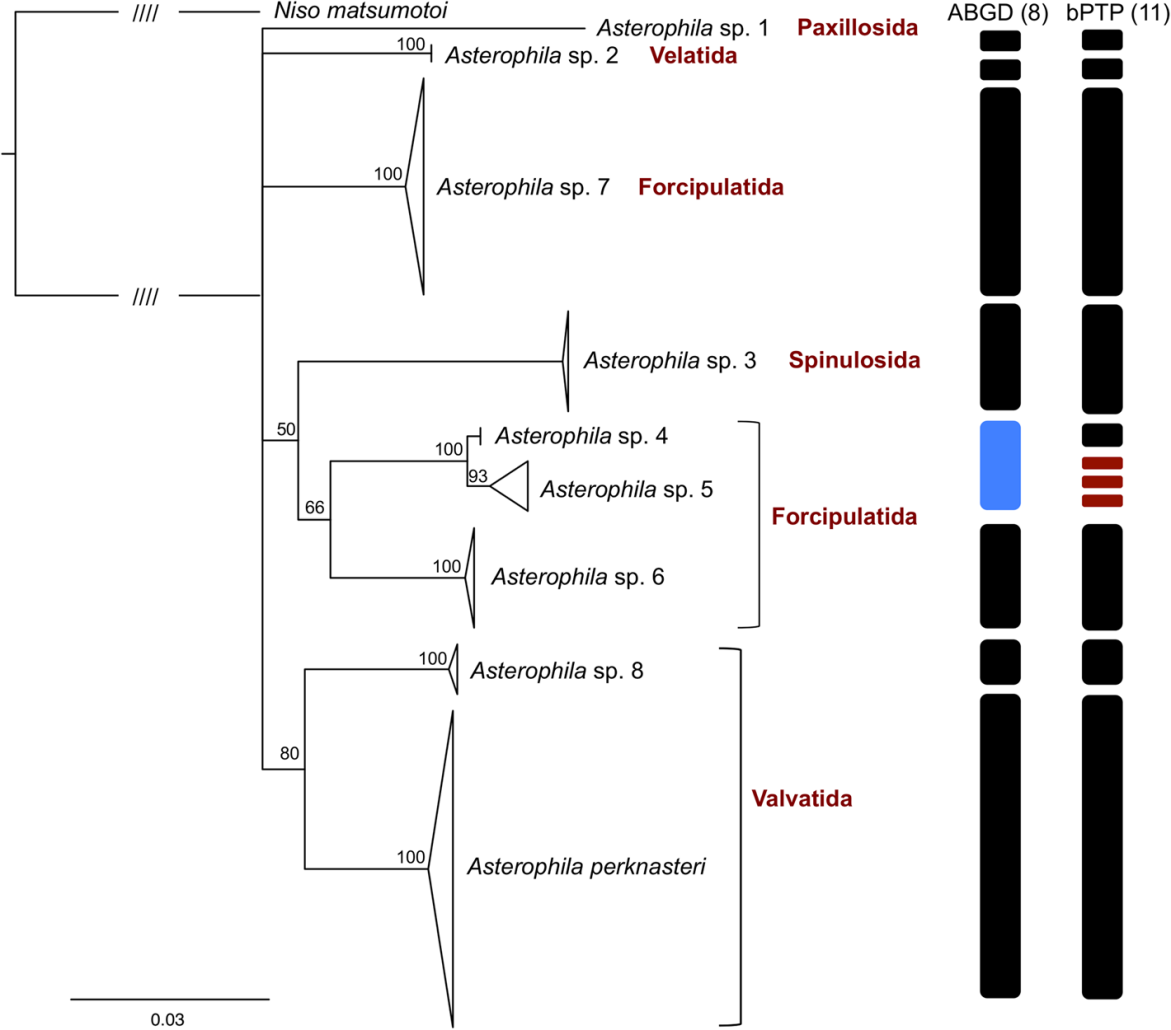
Secondary species hypothesis. ML phylogeny (COI + 16S + H3 + 28S + ANT) of *Asterophila* representing the secondary species hypothesis, with host order-level taxonomy provided in red. Nodes with less than 50% bootstrap support have been collapsed, and triangles represent single clades. Hash marks denote that the branch has been truncated to one quarter of its original length. For species-delimitation analysis (ABGD and bPTP) black boxes represent congruent clades, red boxes represent splits within a clade, and blue boxes represent merged clades

A total of 58 COI, 41 16S, 49 H3, 38 28S, and 14 ANTsequences were recovered from 61 *Asterophila* specimens and concatenated for phylogenetic analysis with a final alignment of 4150 bp (COI: 658 bp; 16S: 455 bp; H3: 328 bp; ANT: 499 bp; 28S: 2210 bp) and 477 variable sites. The COI, 16S, and ANT markers were more variable among species than H3 and 28S. Despite some instances of low bootstrap support at interior nodes, there was still strong support for nine putative species-level entities within *Asterophila* (BS 93–100), forming the PSH (Additional file 1: Figure S1). Each of these nine putative species form highly supported least-inclusive monophyletic clades in the phylogeny and were found in different host families, further supporting their status as separate species. The ML results of the bPTP analysis partitioned *Asterophila* into 11 species, while the initial partition of ABGD delimited 8 species, suggesting over-splitting in the former and merging in the later (Fig. 2). However, the two methods were congruent regarding all but two lineages (sp. 4 and 5). Because each of the clades identified in the PSH were included in one of the species delimitation algorithms, the SSH (Fig. 2) remains the same as the PSH (Additional file 2: Figure S2). One species was identified as *A. perknasteri*, currently known from Antarctica, and the remaining eight were assigned interim names (i.e. *A.* sp. 1- *A.* sp. 8)(Fig. 2). Some sister-species relationships within *Asterophila* were resolved, including *A. perknasteri* and *A.* sp. 8 from the asteroid order Valvatida and *A.* sp. 3, *A.* sp. 4 and *A.* sp. 5 from Forcipulatida, although the position of *A.* sp. 7, also from Forcipulatida, was not well supported (Fig. 2). The positions of *A.* sp. 1 and *A.* sp. 2 were also not well supported in this analysis. Interspecific distances for COI ranged from a minimum of 1.9% between *A.* sp. 4 and *A.* sp. 5 and a maximum of 18.3% between *A.* sp. 1 and *A.* sp. 2 (mean = 13.9%), while intraspecific distances ranged from 0 to 0.9%. An ancestral state reconstruction suggests that a Forcipulatida asteroid is the ancestral host for *Asterophila* (likelihood 68), with one transition to each of Paxillosida, Spinulosida, Valvatida and Velatida (Additional file 2: Figure S2), although a fully resolved phylogeny with additional genes would aid in clarifying these patterns.

This study presents COI sequences for 35 out of 38 individual asteroid hosts with a final alignment of 700 bp. Three hosts missing molecular data were excluded from cophylogenetic analyses, including two *Labidiaster annulatus* and one *Perknaster.* Because there are currently seven species of *Perknaster* known from Antarctic waters (http://www.scarmarbin.be) the species-level identity of this host could not be validated. In all, we report *Asterophila* from five orders, seven families, ten genera, and 15 species of asteroid sea star.

**Fig. 3:**
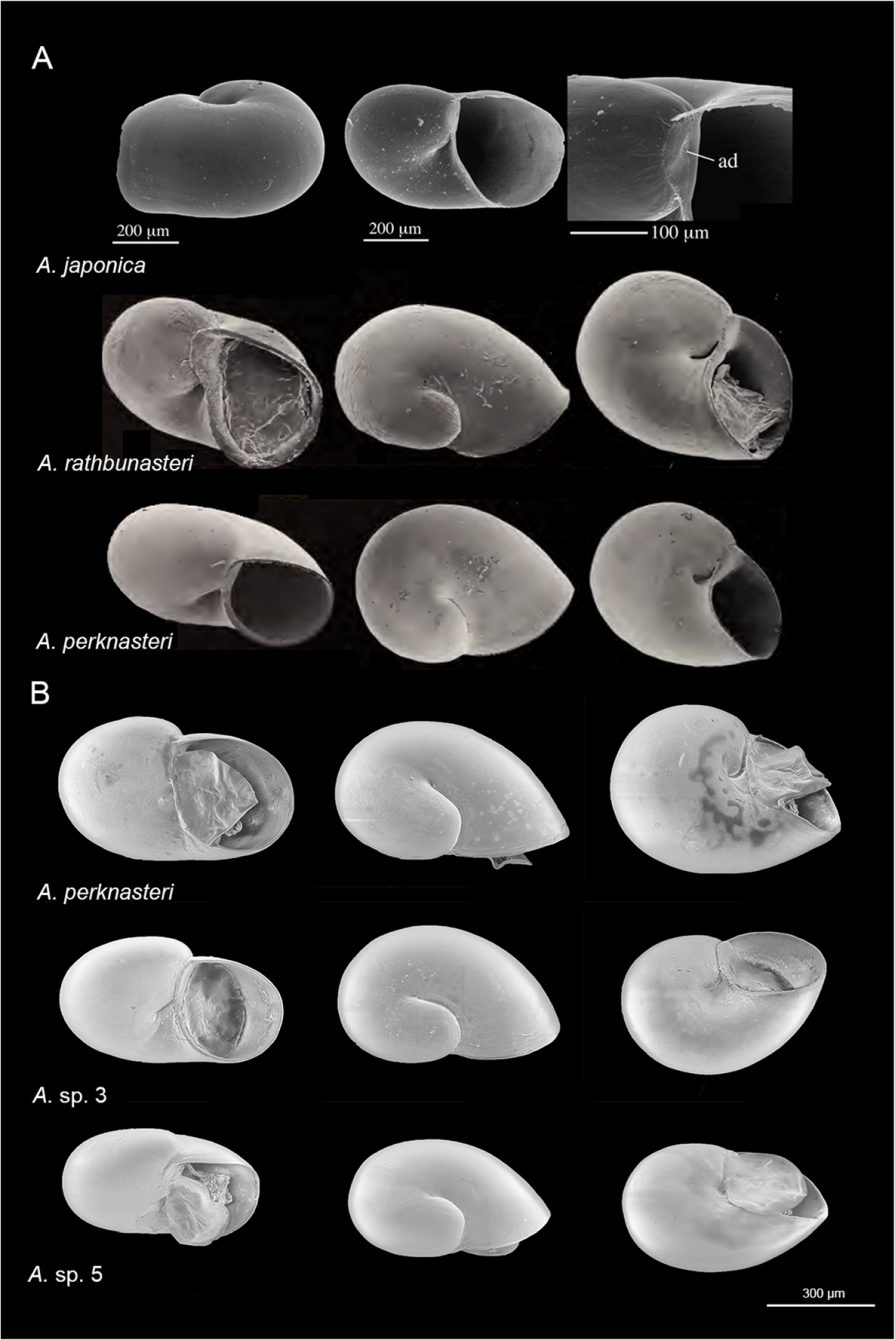
*Asterophila* larvae. Larval morphologies of **a** *Asterophila japonica*, *A. rathbunasteri* and *A. perknasteri* from the literature (adapted from [15, 16] with permission) and **b** *A.* sp. 3, *A.* sp. 5 and *A. perknasteri* from this study, with scale bar

Veliger larvae were recovered from individuals of three *Asterophila* species (*A. perknasteri*, *A.* sp. 3, *A.* sp. 5) and a total of nine larvae were examined and compared to larval morphologies in the existing literature (Fig. 3) (e.g. [15, 16]). From initial observation of SEM images, the larval shells of *A.* sp. 3 and *A.* sp. 5 appear more depressed than the globose shape of *A. perknasteri*. The size of the larval shell was significantly different among species (*P* = 0.002) and measured 690–720 μm in *A. perknasteri*, 650–690 μm in *A.* sp. 3, and 540–600 μm in *A.* sp. 5. The shell width reported for *A. perknasteri* in this study corresponds to the size range reported in the original description of this species (650–760 μm) [15]. Moreover, the umbilicus of *A. perknasteri* appears slightly deeper than both *A.* sp. 3 and *A.* sp. 5, although this character is not mentioned in the existing *Asterophila* descriptions. The larval shells of all species examined in this study appear smooth, with no sculpturing.

### Host specificity and partitioning

**Fig. 4:**
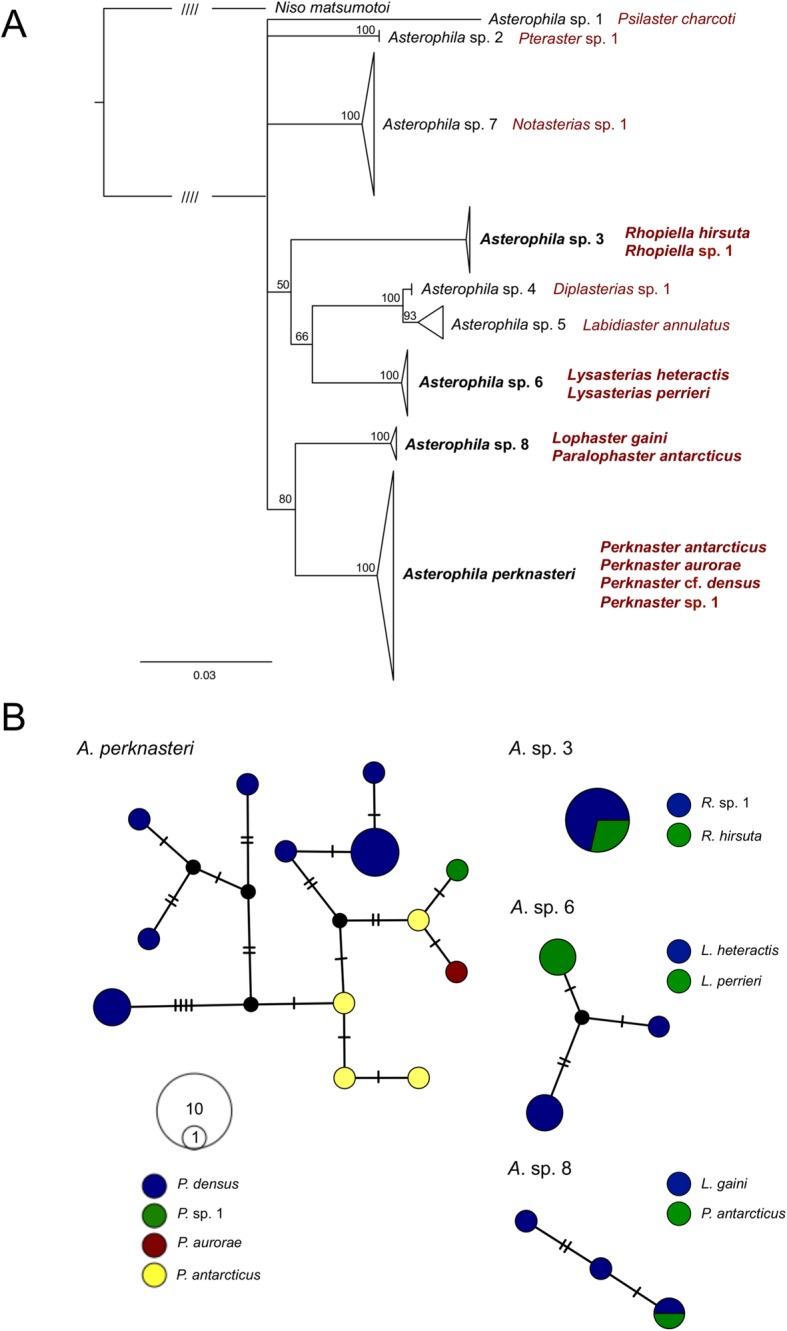
Host use by *Asterophila*. **a** ML phylogeny (COI + 16S + H3 + 28S + ANT) of *Asterophila* with host species designations. Nodes with less than 50% bootstrap support have been collapsed, and triangles represent single clades. Hash marks denote that the branch has been truncated to one quarter of its original length. *Asterophila* species in bold have been recovered from multiple host species. **b** Corresponding TCS haplotype networks (COI) for *Asterophila* species recovered from multiple hosts. Hash marks on the haplotype network correspond to mutational steps and circle size represents the number of sequences per haplotype. Colours in the haplotype network correspond to different host species

*Asterophila* was discovered in 38 hosts from a wide taxonomic breadth. Phylogenetic conservatism in host-use and host-specificity was observed in this system, with eight putative *Asterophila* species each discovered from a different host genus and one species discovered from two host genera (Fig. 4a; Additional file 2: Figure S2). Four species of *Asterophila* (*A.* sp. 3, *A.* sp. 6, *A.* sp. 8, *A. perknasteri*) were found on multiple hosts, but these hosts were always closely related congeners (i.e. *Lysasterias heteractis*, *Lysasterias perrieri*) or confamilials (i.e. *Lophaster gaini*, *Paralophaster antarcticus*) (Fig. 4a), confirmed by significantly lower cophenetic distances between these host pairs than between all other hosts (*P* < 0.0001). Those species found parasitizing multiple hosts also had some of the largest sample sizes (*N* = 7 for *A.* sp. 3, *N* = 7 for *A.* sp. 6, *N* = 4 for *A.* sp. 8, *N* = 20 for *A. perknasteri*). The haplotype networks show some level of host partitioning for *A.* sp. 6 and *A. perknasteri,* demonstrated by a lack of haplotype sharing between individuals from different hosts and some clustering of haplotypes based on host species (Fig. 4b). Individual hosts were only ever parasitized by a single *Asterophila* species.

### Cophylogenetic analysis

**Fig. 5:**
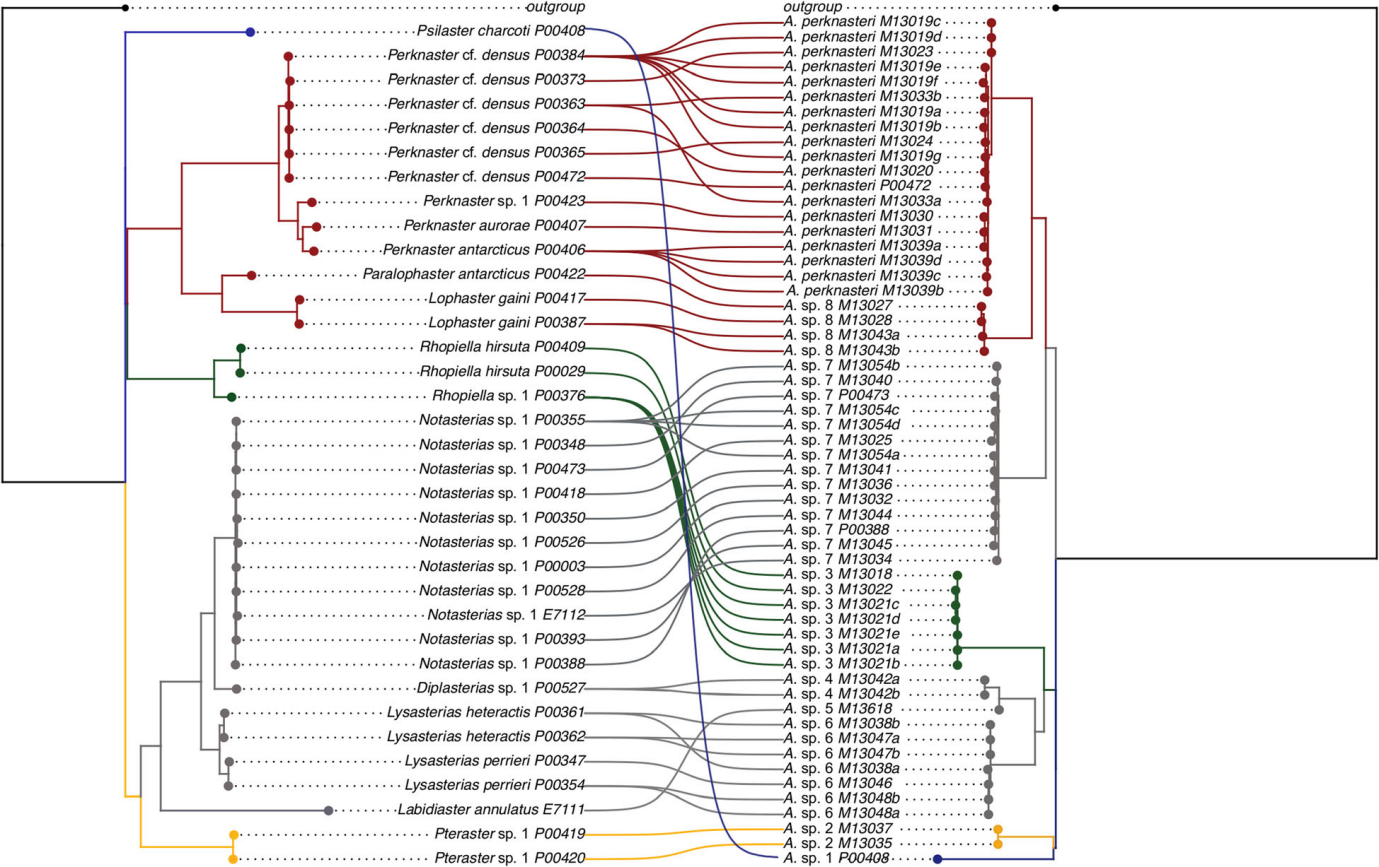
Host-parasite associations. Cophylo plot of 57 host-parasite relationships between *Asterophila* and asteroid hosts. Branches and associations are coloured based on host order. Host colours match ancestral state reconstruction in Additional file 2: Figure S2

A total of 57 *Asterophila* and 35 asteroid hosts were included in the cophylogenetic analysis, with the resulting cophylogenetic plot showing 57 individual associations (Fig. 5). Three specimens of *A.* sp. 5 from *Labidiaster annulatus* and one specimen of *A. perknasteri* from *Perknaster* were excluded from the cophylogenetic analysis because sequence data was unavailable for the host. The event-based analysis in Jane was conducted with four different cost regimes (Table 1), and in all cases the number of non-cospeciation events was greater than the number of cospeciation events, with the number of host-switching events particularly high in the equal costs regime and when duplication and host switches were assigned values of 0. The total cost using 50 random parasite trees was significantly greater than the costs recovered for two of the regimes using the real dataset (*P* < 0.0001) (Table 1), suggesting a strong signal of coevolution in this system. There was no variation in costs using the random parasite trees or the real dataset for the equal costs regime and when duplications and host switches were assigned values of 0. The distance-based analysis in ParaFit revealed that a global test of coevolution was strongly significant (*P* = 0.0001), rejecting the null hypothesis of random association (i.e. that hosts and parasites are evolving independently). A test of coevolution for the 57 associations with a Bonferroni correction revealed a total of 44 significant links (*P* < 0.05) (Table 2).

**Table 1:** Results of event-based reconciliation analysis in Jane

Regime	Cospeciation	Duplication	Host-switch & duplication	Loss	Failure	*p*-value
11111	0	13	43	0	0	n/a
01211	18	7	31	10	0	< 0.0001
11211	6	18	32	1	0	< 0.0001
10011	0	13	43	0	0	n/a

The total number and cost is provided for each coevolutionary event. The total number of cospeciation and non-cospeciation events is also provided. *P*-values were obtained by comparing total cost of the host/parasite phylogenies to the total cost with a random parasite tree

**Table 2:** Results of distance-based analysis in ParaFit

Host Species	Parasite Species	F1 statistic	*p*-value
*Psilaster charcoti* P00408	*Asterophila* sp. 1 P00408	0.0002	0.161
*Rhopiella hirsuta* P00029	*Asterophila* sp. 3 M13022	− 0.0008	0.489
*Rhopiella hirsuta* P00409	*Asterophila* sp. 3 M13018	− 0.0008	0.489
*Rhopiella* sp. 1 P00376	*Asterophila* sp. 3 M13021b	−0.0008	0.488
*Rhopiella* sp. 1 P00376	*Asterophila* sp. 3 M13021c	−0.0008	0.487
*Rhopiella* sp. 1 P00376	*Asterophila* sp. 3 M13021d	−0.0008	0.488
*Rhopiella* sp. 1 P00376	*Asterophila* sp. 3 M13021e	−0.0008	0.489
*Rhopiella* sp. 1 P00376	*Asterophila* sp. 3 M13021a	−0.0008	0.489
***Lophaster gaini*** **P00387**	***Asterophila*** **sp. 8 M13043b**	**0.0010**	**0.022**
*Lophaster gaini* P00387	*Asterophila* sp. 8 M13043a	−0.0013	0.500
*Lophaster gaini* P00417	*Asterophila* sp. 8 M13028	−0.0015	0.500
*Paralophaster antarcticus* P00422	*Asterophila* sp. 8 M13027	−0.0014	0.500
***Perknaster*** **sp. 1 P00423**	***Asterophila perknasteri*** **M13030**	**0.0036**	**0.0002**
***Perknaster antarcticus*** **P00406**	***Asterophila perknasteri*** **M13039b**	**0.0029**	**0.0003**
***Perknaster antarcticus*** **P00406**	***Asterophila perknasteri*** **M13039a**	**0.0029**	**0.0002**
***Perknaster antarcticus*** **P00406**	***Asterophila perknasteri*** **M13039d**	**0.0029**	**0.0002**
***Perknaster antarcticus*** **P00406**	***Asterophila perknasteri*** **M13039c**	**0.0029**	**0.0003**
***Perknaster aurorae*** **P00407**	***Asterophila perknasteri*** **M13031**	**0.0030**	**0.0004**
***Perknaster*** **cf.** ***densus*** **P00384**	***Asterophila perknasteri*** **M13019f**	**0.0007**	**0.011**
***Perknaster*** **cf.** ***densus*** **P00384**	***Asterophila perknasteri*** **M13019e**	**0.0007**	**0.011**
***Perknaster*** **cf.** ***densus*** **P00384**	***Asterophila perknasteri*** **M13019 g**	**0.0018**	**0.007**
***Perknaster*** **cf.** ***densus*** **P00384**	***Asterophila perknasteri*** **M13019b**	**0.0016**	**0.001**
***Perknaster*** **cf.** ***densus*** **P00384**	***Asterophila perknasteri*** **M13019a**	**0.0016**	**0.001**
***Perknaster*** **cf.** ***densus*** **P00384**	***Asterophila perknasteri*** **M13019d**	**0.0015**	**0.001**
***Perknaster*** **cf.** ***densus*** **P00384**	***Asterophila perknasteri*** **M13019c**	**0.0015**	**0.001**
***Perknaster*** **cf.** ***densus*** **P00472**	***Asterophila perknasteri*** **P00472**	**0.0021**	**0.0004**
***Perknaster*** **cf.** ***densus*** **P00365**	***Asterophila perknasteri*** **M13024**	**0.0021**	**0.0003**
***Perknaster*** **cf.** ***densus*** **P00364**	***Asterophila perknasteri*** **M13020**	**0.0011**	**0.043**
***Perknaster*** **cf.** ***densus*** **P00363**	***Asterophila perknasteri*** **M13033b**	**0.0008**	**0.034**
***Perknaster*** **cf.** ***densus*** **P00363**	***Asterophila perknasteri*** **M13033a**	**0.0009**	**0.029**
***Perknaster*** **cf.** ***densus*** **P00373**	***Asterophila perknasteri*** **M13023**	**0.0009**	**0.028**
***Pteraster*** **sp. 1 P00419**	***Asterophila*** **sp. 2 M13037**	**0.0006**	**0.045**
***Pteraster*** **sp. 1 P00420**	***Asterophila*** **sp. 2 M13035**	**0.0007**	**0.021**
***Lysasterias perrieri*** **P00354**	***Asterophila*** **sp. 6 M13048b**	**0.0006**	**0.041**
***Lysasterias perrieri*** **P00354**	***Asterophila*** **sp. 6 M13048a**	**0.0006**	**0.042**
***Lysasterias perrieri*** **P00347**	***Asterophila*** **sp. 6 M13046**	**0.0006**	**0.040**
***Lysasterias heteractis*** **P00362**	***Asterophila*** **sp. 6 M13047b**	**0.0007**	**0.039**
***Lysasterias heteractis*** **P00362**	***Asterophila*** **sp. 6 M13047a**	**0.0007**	**0.038**
***Lysasterias heteractis*** **P00361**	***Asterophila*** **sp. 6 M13038b**	**0.0007**	**0.038**
***Lysasterias heteractis*** **P00361**	***Asterophila*** **sp. 6 M13038a**	**0.0007**	**0.038**
***Diplasterias*** **sp. 1 P00527**	***Asterophila*** **sp. 4 M13042a**	**0.0007**	**0.037**
***Diplasterias*** **sp. 1 P00527**	***Asterophila*** **sp. 4 M13042b**	**0.0006**	**0.036**
***Notasterias*** **sp. 1 P00473**	***Asterophila*** **sp. 7 P00473**	**0.0010**	**0.009**
***Notasterias*** **sp. 1 P00393**	***Asterophila*** **sp. 7 M13045**	**0.0009**	**0.008**
***Notasterias*** **sp. 1 P00418**	***Asterophila*** **sp. 7 M13025**	**0.0010**	**0.007**
***Notasterias*** **sp. 1 P00003**	***Asterophila*** **sp. 7 M13032**	**0.0010**	**0.007**
***Notasterias*** **sp. 1 P00528**	***Asterophila*** **sp. 7 M13044**	**0.0010**	**0.008**
***Notasterias*** **sp. 1 P00526**	***Asterophila*** **sp. 7 M13036**	**0.0009**	**0.009**
***Notasterias*** **sp. 1 P00350**	***Asterophila*** **sp. 7 M13041**	**0.0010**	**0.008**
***Notasterias*** **sp. 1 P00348**	***Asterophila*** **sp. 7 M13040**	**0.0009**	**0.008**
***Notasterias*** **sp. 1 P00388**	***Asterophila*** **sp. 7 P00388**	**0.0009**	**0.011**
***Notasterias*** **sp. 1 P00355**	***Asterophila*** **sp. 7 M13054c**	**0.0012**	**0.003**
***Notasterias*** **sp. 1 P00355**	***Asterophila*** **sp. 7 M13054d**	**0.0012**	**0.003**
***Notasterias*** **sp. 1 P00355**	***Asterophila*** **sp. 7 M13054a**	**0.0014**	**0.0003**
***Notasterias*** **sp. 1 P00355**	***Asterophila*** **sp. 7 M13054b**	**0.0014**	**0.0003**
*Notasterias* sp. 1 E7112	*Asterophila* sp. 7 M13034	0.0002	0.154
*Labidiaster annulatus* E711	*Asterophila* sp. 5 M13618	0.0002	0.156

Tests of individual congruence between host and parasite and the corresponding *p*-value. Associations in bold are significant (*P* < 0.05). Global congruence analysis in ParaFit was significant (ParaFitGlobal = 0.03; *P* = 0.0001)

## Discussion

### Phylogeny and diversification of *Asterophila* 

This study uncovers a radiation of *Asterophila* in Antarctica and increases the diversity of this group nearly tenfold, from one previously known species in the region (*A. perknasteri*) to a putative nine species. Although each of these putative nine species-level entities was strongly supported in the ML phylogeny, deeper relationships remain poorly supported. A lack of support in the *Asterophila* phylogeny, despite the use of both mitochondrial and nuclear markers that should be informative at both shallow and deep divergences, suggests that additional taxonomic and genomic sampling is required. Nevertheless, molecular data has been crucial for delimiting species boundaries in this genus. Each putative *Asterophila* species was recovered from a different host family, suggesting that host identity also serves as an informative character for species delimitation. The COI divergence between *A.* sp. 4 and *A.* sp. 5 is relatively low (1.9%) compared to interspecific distances for other molluscs (e.g. [29, 30]), but because these species were separated in the bPTP analysis and were recovered from different host families they are retained as different lineages in this study. Additional specimens suitable for molecular and morphological analysis would aid in clarifying these species boundaries. In all, this study reveals a novel diversification of endoparasites in Antarctica and extends our knowledge of diversity in *Asterophila*.

Previous work by Sasaki et al. [16] used larval shell characters to diagnose three known species of *Asterophila*. These characters include an apical depression on the protoconch of *A. japonica*, rugose sculpture and a less depressed protoconch in *A. rathbunasteri*, and a large, smooth larval shell in *A. perknasteri*. The larval shells of two undescribed species examined in this study (*A.* sp. 3, *A.* sp. 5) appeared more similar in general shape to *A. perknasteri* than to either *A. japonica* or *A. rathbunasteri* but had a shallower umbilicus and appeared more depressed. Although larval shell size appeared to differ significantly between three Antarctic *Asterophila* species examined in this study, this variation in size may be related to differences in developmental stage and age of the larvae when they were retrieved from adult females. As such, there exists a deficit of informative morphological characters in this endoparasitic genus. Recent studies have also highlighted difficulties in using morphological characters for species delimitation in highly modified endoparasites (e.g. splanchnotrophid copepods [24, 31]), and this work further demonstrates that molecular data is critical for resolving species boundaries in endoparasites that often lack informative external characters.

Some benthic marine invertebrate groups have radiated extensively in Antarctica, including isopods [32], dorid nudibranchs [4, 33], octopods [34], and pycnogonids [35], and repeated glacial cycles and the subsequent fragmentation of populations has been important in promoting speciation in these marine taxa [3]. Glacial and interglacial cycles have presumably had an impact on the diversity and distribution of *Asterophila* in the region. The retreat of populations into small, closely distributed glacial refugia around Antarctica would have facilitated host-switching events by bringing multiple potential hosts into proximity, as well as by driving some hosts to extinction [13]. It is also likely that the close coupling of endoparasite and host has been important in driving the diversification of this group.

### Patterns of coevolution between *Asterophila* and asteroid hosts

Coevolution and cospeciation are often used synonymously in the literature, but the former simply refers to reciprocal evolution between two or more species and can involve several processes, mainly cospeciation, duplication, host-switching, loss of a lineage, or failure of a parasite to diverge [36]. Although a distance-based analysis in ParaFit sheds light on a signal of cospeciation in the system, the event-based analysis in Jane reconciles the specific events explaining the host-parasite associations. Both the event-based and distance-based analysis showed a global signal of coevolution, thereby rejecting the null hypothesis of independent evolution between host and parasite. A total of 30 links were significant in the ParaFit analysis (*P* < 0.05), with an increase to 44 following a Bonferroni correction. These results suggest a strong signal of coevolution, likely driven by host-switching speciation events that have produced congruent phylogenies, but the parasite phylogeny was poorly supported and the host phylogeny was based on a single mitochondrial marker. The different cost regimes in Jane produced different numbers of events, most notably ranging from zero cospeciation events in the first regime to 18 cospeciation events in Jane’s default regime, suggesting that some regimes under-detect cospeciation events and that choosing a regime a priori may prove problematic. In any case, most events were explained by duplication and host-switching, suggesting that non-cospeciation events are primarily driving the coevolution of *Asterophila* and their hosts.

Recent work has highlighted the importance of non-cospeciation events in the diversification of endoparasites (e.g. endoparasitic nematodes and stick insect hosts [23]) and in highly specialized symbionts (e.g. feather mites and birds [37, 38]), demonstrating that these obligate host-parasite systems do not always follow strict cospeciation and synchronous evolution [39]. Host-switching events are thought to be of central importance in driving parasite diversity (e.g. [40, 41]), and several studies have shown that host switches are particularly important in the diversification of Antarctic parasites, suggesting that repeated glacial cycles in the region fragmented populations and differentially removed potential hosts [12, 13]. Alternatively, the high level of host specificity and host switching observed in this system, and the sympatric distribution of most parasites and hosts, could indicate that parasites are able to diversify and adapt to new hosts in sympatry, rather than through allopatric speciation (e.g. [24]).

Several additional factors may also have played a role in promoting host-switching in endoparasitic *Asterophila*, including the presence of a planktonic larval phase, which results in greater dispersal potential and the ability to encounter new hosts [40], and the sympatric distribution of host species allowing for more host-switching opportunities (e.g. [42]), with multiple hosts of *Asterophila* co-occurring in Antarctica. The mechanism by which *Asterophila* larvae locate and occupy a host is currently unknown, but chemical cues from hosts induce settlement of planktonic larvae in other molluscs (e.g. coral-associated nudibranchs [43]) and are likely integral to this system. Furthermore, the high diversity of *Asterophila* species uncovered in this study may also be linked to host-switching, as the isolation of parasites on new hosts can promote their diversification (e.g. [38, 39, 41]). In all, non-cospeciation events explained most of the associations between *Asterophila* and their hosts, highlighting the importance of these events even in endoparasitic lineages.

Most *Asterophila* species in this study were discovered from a single host genus, and always from a single host family, which differs from other endoparasites, including a species of splanchnotrophid copepod (*Lomanoticola brevipes*) that was recovered from five different nudibranch families [24]. However, incongruence between the parasite and host phylogenies in this study implies that this specificity varies among species [42]. For instance*, A.* sp. 1, 2, 4, 5, and 7 were all recovered from a single host species, while *A.* sp. 3, *A.* sp. 6, and *A. perknasteri* were recovered from two to four congeners, and *A.* sp. 8 was recovered from two genera in Solasteridae. The haplotype networks for *A.* sp. 6 and *A. perknasteri* show a complete lack of haplotype sharing between individuals from different hosts, indicating potential host partitioning and incipient host-shift speciation, but additional samples are needed to validate these patterns. In any case, multihost *Asterophila* species were recovered from phylogenetically-related hosts, as demonstrated by lower cophenetic distances between host pairs than between all other hosts, and these hosts often had the highest sample size, illustrating that the recovery of hosts, and ultimately parasite richness, may be linked to sampling effort (e.g. [44, 45]).

Poulin [42] suggests that a pattern of host generalism is often shown in parasites that are recovered from i) species-rich host groups and ii) hosts with unstable or fluctuating populations, both of which are true of the Antarctic echinoderm fauna and may explain multihost use in *Asterophila*. Both the ML phylogeny and transformation suggest phylogenetic conservatism of host use, although further resolution of the *Asterophila* phylogeny, possibly through the use of a reduced representation library, will be crucial to better understanding these patterns. Phylogenetic conservatism of host use has been shown in other marine parasites, including in myzostomid annelids and their echinoderm hosts [22]. An ancestral state reconstruction also suggests that a member of Forcipulatida was the ancestral host to *Asterophila*, but with only moderate support [46]. Continued sampling will undoubtedly enable the recovery of additional hosts, and other *Asterophila* species, which will aid in clarifying their coevolutionary relationships. Future work should also look to sample from greater depths as *Asterophila* has yet to be reported from two orders of deep-sea asteroids. Lastly, previous studies have suggested that polyparasitism, where hosts are infected by multiple parasite species, is prevalent in natural populations [47–49], but this study recovered only a single *Asterophila* species from each host individual.

### Antarctic distribution of *Asterophila* 

Combining this study and the original description of *A. perknasteri* [15], *Asterophila* has now been reported from the South Shetland Islands, Elephant Islands, South Orkney Islands, South Georgia, and Shag Rocks, comprising the tip of the Antarctic Peninsula and parts of the Scotia Arc. Eight putative *Asterophila* species in this study were found co-distributed along the South Shetland Islands and Elephant Island, and a ninth putative species was found at Shag Rocks and South Georgia, but the absence of a single *Asterophila* species at both locations suggests their ranges are restricted. The octopus *Pareledone charcoti* also appears restricted to the South Shetland Islands and although this distribution may be due to deep-water and currents restricting gene flow in the area, it is also a consequence of a lack of a planktonic larval phase [50], which *Asterophila* does possess. A single host with parasitic cyst was imaged from South Orkney but this specimen was not recovered from the collection for analysis. This host appears to be *Diplasterias* however, suggesting that the accompanying parasite could be *Asterophila* sp. 4. If so, this would extend the distribution of *A.* sp. 4 from the northern Shetland Islands and Elephant Island into South Orkney Islands, although additional samples and molecular analysis are needed to confirm this result. Moreover, some marine taxa show distinct genetic differences between populations at Shag Rocks and South Georgia (e.g. mackerel icefish [51], octopus *Pareledone turqueti* [52]), despite a short distance between them, but only four specimens of *A.* sp. 5 were retrieved from Shag Rocks and South Georgia in this study and therefore additional sampling is needed before elucidating any phylogeographic patterns. Shag Rocks has also been declared a biodiversity hotspot for benthic marine invertebrates [53], warranting further investigation in this area.

The distribution of parasite depends on the distribution of its host [42], but a plethora of potential hosts are distributed around Antarctica and the subantarctic islands and thus the absence of *Asterophila* at many locations outside the Scotia Arc is surprising. Salmen et al. [54] uncovered a similar pattern in endoparasitic copepods, where parasites were only recovered from Sulawesi, Indonesia despite nudibranch hosts being broadly distributed across the tropical Indo-Pacific. The restricted co-distribution of *Asterophila* may also indicate that host-specific parasites are able to adapt to new hosts in sympatry, as suggested by Anton et al. [24]. However, the reported distribution of *Asterophila* in this study could also be an artefact of concentrated sampling around the Antarctic Peninsula and Scotia Arc, combined with a low prevalence of parasitism in this group. For instance, Sasaki et al. [16] found the rate of *A. japonica* infection ranged from just 2.2 to 17.5% in Japan, and Schiaparelli et al. [12] found similarly low rates of infection by the eulimid *Bathycrinola tumidula* on the crinoid *Notocrinus virilis* in Antarctica. Given that hundreds of asteroids were examined in this study, and parasites were only discovered from 38 hosts, one could assume that the prevalence of parasitism in this system is comparably low and thus intensive sampling will be required for a more complete understanding of the distribution of this genus in Antarctica. Sampling effort at present has been focused around the Antarctic Peninsula and Scotia Arc, likely skewing the reported distribution of *Asterophila* in this study. In fact, specialist host-parasite systems rarely exhibit a broad distribution [46], so it is likely that continued sampling around Antarctica, particularly in east Antarctica, will uncover a new suite of *Asterophila* species. Alternatively, the use of different trawling equipment among research cruises may have negatively impacted host and parasite recovery in this study, ultimately skewing rates of parasitism. Lastly, although adult *Asterophila* form large, noticeable cysts in asteroid seastars, it is likely that recently settled juveniles would be less detectable in hosts. In any case, the marine fauna of the Antarctic Peninsula and surrounding subantarctic islands has been shown to be incredibly diverse (e.g. [7–9]), and this study reveals this area may also be a ‘coevolutionary hotspot’, although these results may also point to a general lack of knowledge and study of parasite diversity on a global scale. As annual shelf temperatures in this region continue to warm (e.g. [55]) the conditions are expected to promote range expansions in marine taxa [56], potentially affecting the distribution of *Asterophila* through expansions in host range. Moreover, given the threat of coextinction to the ongoing biodiversity crisis (e.g. [57]), coupled with accelerated rates of climate change in the region, it is crucial that we continue to document and understand host-parasite diversity in Antarctica.

## Conclusions

This is the first study addressing Antarctic *Asterophila* since the initial description in 1994, uncovering eight putative new species, adding several new host genera, and expanding its distribution from the tip of the Antarctic Peninsula to Shag Rocks and South Georgia. This work also explores cophylogenetic patterns between *Asterophila* and their hosts, demonstrating that non-cospeciation events are primarily driving diversification in this system. Four of nine *Asterophila* species were found on multiple hosts, but these hosts were always phylogenetically related, being from one or two genera from the same family. Relationships between *Asterophila* species remain poorly resolved and obtaining molecular data for other congeners will be essential to better understand interspecific relationships in this genus. Additionally, a more robust understanding of the life history of these animals is needed, particularly regarding larval development and dispersal potential as well as the biochemical pathway responsible for host recognition. The distribution of *Asterophila* reported in this study is likely not reflective of its true range, due to both under-sampling in most of Antarctica and a potentially low rate of infection. Intensive sampling is needed in east Antarctica and the deep sea, the latter of which is particularly interesting as *Asterophila* has never been recorded from two orders of deep-sea asteroids. Furthermore, future work should continue to investigate cophylogenetic patterns in marine invertebrates and their parasitic counterparts, as these relationships are currently underrepresented in the literature.

## Methods

### Specimen collection and sequence acquisition

A total of 61 *Asterophila* specimens from 38 asteroid hosts were collected on three cruises (RVIB Nathaniel B. Palmer 11–05 in 2011, FS Polarstern ANT-XXVIII/4 in 2012, RVIB Nathaniel B. Palmer 13–03 in 2013) in Antarctica (Fig. 1; Additional file 3: Table S1). Additional hosts were also collected on the NOAA Antarctic Marine Living Resources (AMLR) cruise in 2009 and the Antarctic Circumnavigation Expedition (ACE) in 2016/2017 (Fig. 1). All *Asterophila* and corresponding hosts were collected with a benthic trawl at depths of 123 to 423 m on the continental shelf. Most specimens were preserved in 90–100% ethanol but some vouchers and veliger larva were fixed in 8% formalin and later transferred to 50–70% ethanol for morphological analysis. Some veliger larva are permanently mounted on SEM stubs at the Western Australian Museum (WAM) and remaining specimens and tissue samples are housed at both the Benthic Invertebrate Collection at Scripps Institution of Oceanography (BIC-SIO) and the Western Australian Museum (WAM). Both host and parasite specimens were identified in the field and these identifications were later confirmed and corrected by comparing cytochrome *c* oxidase subunit I (COI) sequences against the Barcode of Life Data System (BOLD) and NCBI BLAST databases.

Preserved tissue from the pseudopalium of *Asterophila* and tube feet from asteroid hosts were used for DNA extraction with DNeasy spin columns (Qiagen). Some molecular methods employed in this study have been previously described by [58]. Primers used for PCR and sequencing are listed in Table 3. For samples that were difficult to amplify, DNA dilutions (1:100) were used as template in PCR. Each PCR reaction included: 16.8 μl molecular grade water, 5 μl 5x MyTaq PCR buffer (Bioline), 0.5 μl forward and reverse primers (10 μM), and 0.2 μl platinum Taq polymerase (Bioline). The thermocycling regime for COI was: 3 min at 95 °C, 4 cycles of 40 s at 95 °C, 40 s at 45 °C, and 50 s at 72 °C, followed by 35 cycles of 40 s at 95 °C, 40 s at 51 °C, and 50 s at 72 °C, with a final extension for 10 min at 72 °C. Some asteroids were run with the following conditions for COI: 3 min at 95 °C, 8 cycles of 30 s at 95 °C, 30 s at 50 °C, and 45 s at 72 °C, followed by 38 cycles of 30 s at 95 °C, 30 s at 48 °C, and 45 s at 72 °C, with a final extension for 5 min at 72 °C. The thermocycling regime for 16S ribosomal RNA (16S), histone 3 (H3), and 28S ribosomal RNA (28S) was: 2 min at 94 °C, 35 cycles of 30 s at 94 °C, 40 s at 50 °C, and 60 s at 72 °C, with a final extension for 10 min at 72 °C. For some 28S reactions that failed in the first round of amplification, the following touch-down thermocycling regime was used in the second round; 2 min at 94 °C, 10 cycles of 30 s at 60 °C and decreasing by 0.5 °C every cycle until a final annealing temperature of 55.5 °C, 20 cycles of 30 s at 94 °C, 40 s at 55 °C, and 60 s at 72 °C, with a final extension for 10 min at 72 °C. The adenine nucleotide translocase (ANT) R1 primer was used for initial PCR and 2 μl of diluted (1:100) PCR template from this reaction was used for a second nested PCR with the ANT R2 primer. The thermocycling regime for ANT was: 3 min at 95 °C, 34 cycles of 40 s at 95 °C, 45 s at 51 °C, and 60 s at 68 °C, with a final extension for 5 min at 68 °C. Amplicons were screened on E-gels (Invitrogen) and all positive reactions were bidirectionally sequenced using the chain termination method and Big Dye Terminator kit. Sequencing was outsourced to the Australian Genome Research Facility (Perth). All sequences were edited in Geneious v.8.0.5 and aligned with MAFFT using default settings.

**Table 3:** Primers used for PCR and sequencing in this study

Primer Name (F/R)	Marker	F/R Sequence (5′ to 3′)	Reference
LCO1490/ HCO1490	COI parasites	GGTCAACAAATCATAAAGATATTGG/ TAAACTTCAGGGTGACCAAAAAATCA	[59]
COIceF/ COIceR or jgLCO1490/ jgHCO2198	COI hosts	ACTGCCCACGCCCTAGTAATGATATTTTTTATGGTN-ATGCC/ TCGTGTGTCTACGTCCATTCCTACTGTRAA-CATRTG TITCIACIAAYCAYAARGAYATTGG/ TAIACYTCIGGRTGICCRAARAAYCA	[60, 61]
16SarL/ 16SbrH	16S	CGCCTGTTTATCAAAAACAT/ CCGGTCTGAACTCAGATCACGT	[62]
H3MF/H3MR	H3	ATGGCTCGTACCAAGCAGACTGC/ TGGATGTCCTTGGGCATGATTGTTAC	[63]
ANTF/ANTR1/ ANTR2	ANT	CCATTYTGGMGIGGWAACWTGGC/ TTCATCAAIGACATRAAICCYTC/ CCCTTGTAYTTRACASCYTCACC	[64]
1100F (forward) na2 (reverse) with	28S	GGACCCGAAAGATGGTGAACTATGC/ AGCCAATCCTTATCCCGAAG	[25, 65]
FL (forward)*		AAGTGGAGAAGGGTTCCATGT	[25]
LSU5/ LSU1600R with ECD25 (reverse)*		TAGGTCGACCCGCTGAAYTTAAGCA/ AGCGCCATCCATTTTCAGG CTTGGTCCGTGTTTCAAGACGG	[66, 67] [68]

Multiple primer sets were used to amplify the entire fragment of 28S, with an asterisk denoting sequencing primers

### Phylogenetic reconstruction and species delimitation

*Niso matsumotoi* was selected as an outgroup for *Asterophila* phylogenetic analysis based on a larger phylogeny of Eulimidae (Layton et al. in preparation), and these sequences were obtained from GenBank (AB930469, AB930413, AB930440, AB930335). The phylogenetic methods employed in this study have been previously described by [58]. A dataset comprised of sequences from two mitochondrial genes (COI, 16S) and three nuclear genes (H3, 28S, ANT) was concatenated for subsequent phylogenetic analysis. A maximum likelihood (ML) tree was constructed in RAxML [69] implemented in the raxmlGUI v1.3 [70] using a GTR + G model with partitions set for each gene and 1000 bootstrap replicates calculated with joint partition support. Species were recognized as highly supported least-inclusive clades of terminals in the phylogenetic analysis, forming the primary species hypothesis (PSH) (Additional file 1: Figure S1). A secondary species hypothesis (SSH) was reassessed based on results from two species-delimitation analyses. The tree file was imported into RStudio and the ape package [71] was used to collapse nodes in the SSH with low support (< 50%). Pairwise distances were calculated in MEGA v6.0 using the COI dataset and a maximum composite likelihood model with 100 bootstrap replicates and pairwise deletion. The Bayesian Poisson tree processes (bPTP) [72] and Automated Barcode Gap Discovery (ABGD) [73] algorithms were used to partition the concatenated dataset into unique genetic clusters. The parameters employed for the former were 100,000 MCMC generations, a thinning value of 100, and 10% burn-in, and for the latter were Pmin = 0.001, Pmax = 0.10, 10 steps, X = 1.5, Nb bins of 20, and a Jukes Cantor (JC69) model. The final ML tree was used as an input file for the bPTP analysis and the fasta file for this ML tree was employed for the ABGD analysis. Finally, host type, defined as asteroid order, was mapped onto the uncollapsed ML phylogeny with branch-lengths retained and ancestral state reconstruction was conducted with an Mk1 model in Mesquite 3.04 [74].

### Cophylogenetic analysis

Both distance-based and event-based cophylogenetic analyses were conducted on host and parasite phylogenies. A host phylogeny was generated using COI sequences from asteroids that were found with parasites. The ML tree was generated in RAxML [69] implemented in the raxmlGUI v1.3 [70] using a GTR + G model and 1000 bootstrap replicates. An ophiuroid was used as an outgroup and the topology was constrained to reflect the most current asteroid phylogeny [75]. A corresponding parasite ML phylogeny was generated using only those individuals for which host sequence data was available. The cophylo function in the package Phytools [76] was used in RStudio to visualize host-parasite associations. All individual hosts and parasites were included in subsequent cophylogenetic analysis, but outgroups were excluded. The host and parasite phylogenies were used to construct a tanglegram in TreeMap3.0b [77], which was imported into Jane 4 [78] to run event-based reconciliation analysis with 99,999 permutations, a generation size of 200, and a population size of 100. Random parasite trees were also constructed in Jane with a population size of 50, and a Welch’s two-sample t-test was used to determine whether the costs of each regime using these 50 random parasite trees was different to the costs obtained using real parasite trees. If the cost generated from the random parasite trees is significantly greater than the observed cost then a global signal of coevolution is present [79]. Four different cost regimes were employed in Jane; i) equal costs (11111), ii) assigning cospeciation events a value of 0 (01211), iii) assigning host-switching events a value of 2 (11211), and iv) assigning duplication and host-switching events a value of 0 (10011). Distance-based analysis was run in ParaFit [80] using the ape package [71] in RStudio, which tests for both global and individual congruence. This analysis tests the null hypothesis that hosts and parasites are evolving independently. For ParaFit, cophenetic distances (branch length pairwise differences) were calculated from host and parasite phylogenies using the cophenetic function in the ape package [71] in RStudio, along with an association matrix, 99,999 permutations, and a Cailliez correction for negative eigenvalues. A Bonferroni correction was applied to *p*-values from the ParaFit analysis as two ParaFitLink tests were conducted for each comparison. The Jane and ParaFit analyses were chosen as they allow for uneven numbers of parasites and hosts, including multihost parasites. Lastly, for *Asterophila* species that were discovered from multiple hosts, TCS haplotype networks [81] with COI data and 5000 iterations were generated in PopART [82] for exploring genetic structure and potential host partitioning. To determine whether *Asterophila* species were parasitizing phylogenetically related hosts, cophenetic distances between host pairs (hosts parasitized by the same *Asterophila* species) were compared to cophenetic distances between all other hosts using a Wilcoxon 2-sample rank sum test.

### Larval morphology

Published descriptions of known *Asterophila* species highlight only a few diagnostic morphological characters in this group, some of which are based on larval shell characters [15]. As such, larval shells from four preserved specimens per species were examined using a Hitachi TS3030Plus tabletop scanning electron microscope (SEM) at the Western Australian Museum. Larval shells were transferred directly from 100% ethanol to the SEM stub. The general shape, sculpture and umbilicus were assessed by eye, and larval shell length was measured across the widest point of the shell. A one-way ANOVA was employed in the stats v3.6.0 package [83] in RStudio for comparing mean shell size between species.

## Additional files

Additional file 1: **Figure S1.** Primary species hypothesis. ML phylogeny (COI + 16S + H3 + 28S + ANT) of *Asterophila* with highly supported least inclusive clades (and singletons) representing the PSH. Hash marks denote that the branch has been truncated to one half of its original length. (TIF 474 kb)

Additional file 2: **Figure S2.** Host type**.** Ancestral state reconstruction (ML) for host type using asteroid order and an Mk1 likelihood model. Asterisks mark nodes with a likelihood of > 99%, with values less than this provided at nodes. (TIF 1268 kb)

Additional file 3: **Table S1.** Specimen details. *Asterophila* specimens collected and analysed in this study, along with corresponding host details, locality information, and GenBank accession numbers. Hosts marked with an asterisk lack sequence data. (DOCX 37 kb)

## Data Availability

The datasets used and/or analysed during the current study are available from the corresponding author on reasonable request and GenBank accessions appear in the manuscript.

## Abbreviations

16S: 16S ribosomal RNA
28S: 28S ribosomal RNA
ABGD: Automated Barcode Gap Discovery
ACE: Antarctic Circumnavigation Expedition
AMLR: NOAA Antarctic Marine Living Resources
ANT: Adenine nucleotide translocase
BIC-SIO: Benthic invertebrate Collection at Scripps Institution of Oceanography
BOLD: Barcode of Life Data System
bPTP: Bayesian Poisson tree processes
BS: Bootstrap support
COI: Cytochrome *c* oxidase subunit I
H3: Histone 3
JC69: Jukes Cantor
ML: Maximum likelihood
PSH: Primary species hypothesis
SEM: Scanning electron microscope
SSH: Secondary species hypothesis
WAM: Western Australian Museum
