# Supplementary figures and images for: A newly discovered radiation of endoparasitic gastropods and their coevolution with asteroid hosts in Antarctica
Source: BMC Evol Biol. 2019 Sep 18;19:180. doi: 10.1186/s12862-019-1499-8 (PMC6749685; doi:10.1186/s12862-019-1499-8)

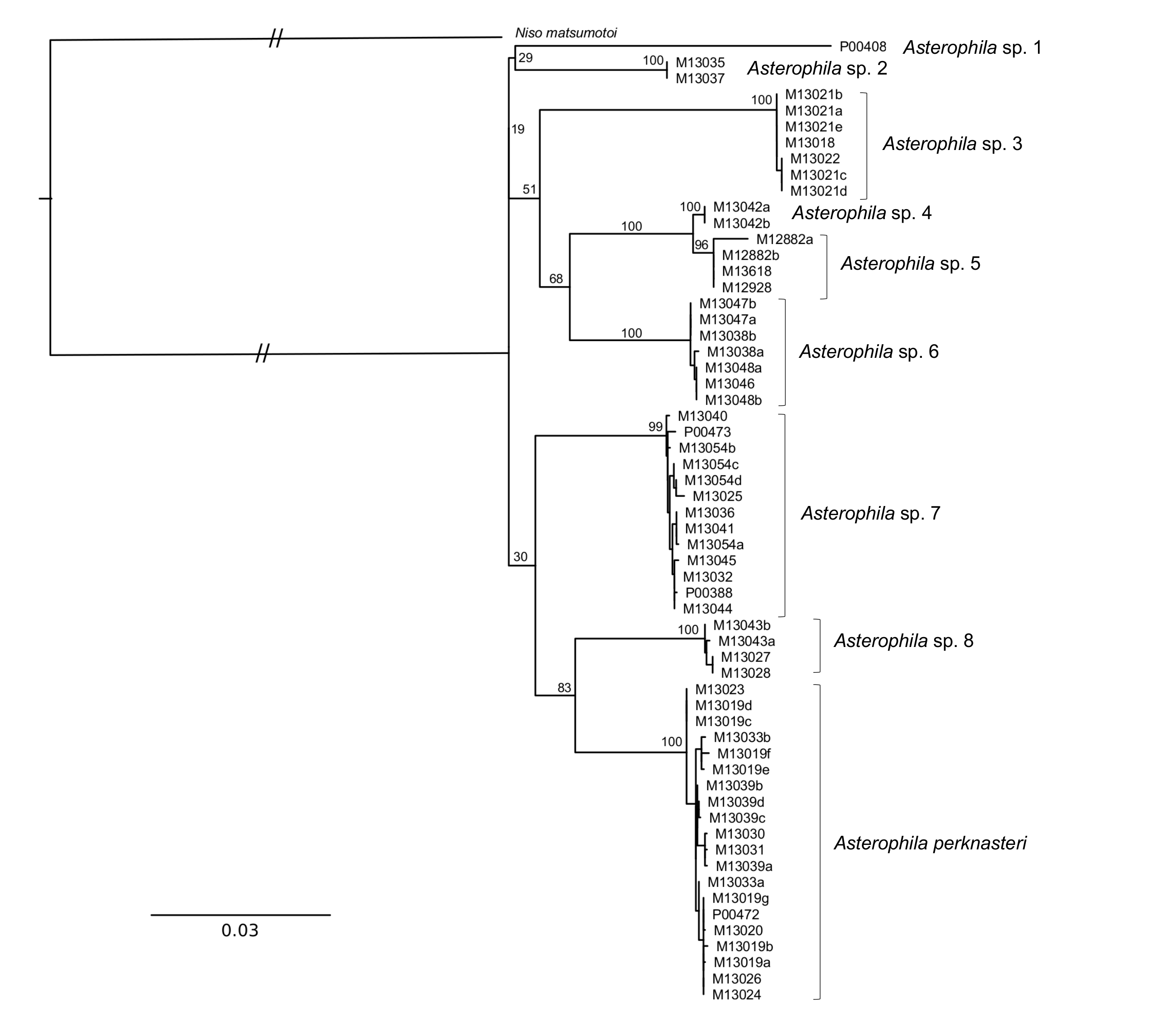

Supplement: Supplementary file 1 — Figure S1. Primary species hypothesis. ML phylogeny (COI + 16S + H3 + 28S + ANT) of Asterophila with highly supported least inclusive clades (and singletons) representing the PSH. Hash marks denote that the branch has been truncated to one half of its original length. (TIF 474 kb) [file 12862_2019_1499_MOESM1_ESM.tif]

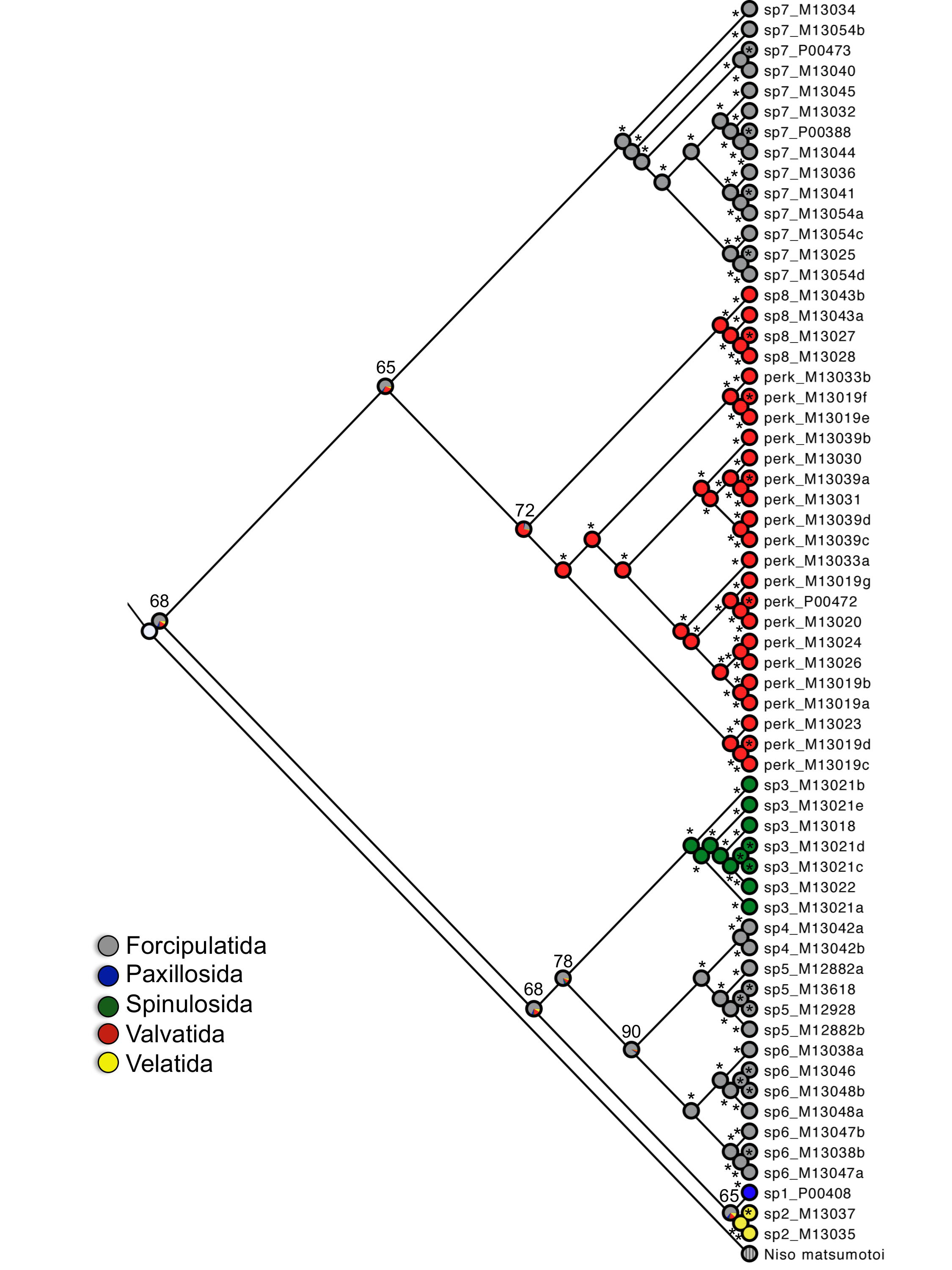

Supplement: Supplementary file 2 — Figure S2. Host type. Ancestral state reconstruction (ML) for host type using asteroid order and an Mk1 likelihood model. Asterisks mark nodes with a likelihood of > 99%, with values less than this provided at nodes. (TIF 1268 kb) [file 12862_2019_1499_MOESM2_ESM.tif]
