# Supplementary material for: A newly discovered radiation of endoparasitic gastropods and their coevolution with asteroid hosts in Antarctica
Source: BMC Evol Biol. 2019 Sep 18;19:180. doi: 10.1186/s12862-019-1499-8 (PMC6749685; doi:10.1186/s12862-019-1499-8)
Supplement: Supplementary file 3 — Table S1. Specimen details. Asterophila specimens collected and analysed in this study, along with corresponding host details, locality information, and GenBank accession numbers. Hosts marked with an asterisk lack sequence data. (DOCX 37 kb) [file 12862_2019_1499_MOESM3_ESM.docx]

**Table S1.** **Specimen details.** *Asterophila* specimens collected and analysed in this study, along with corresponding host details, locality information, and GenBank accession numbers. Hosts marked with an asterisk lack sequence data.

| **Sample ID**  **Parasite** | **Parasite Species** | **Sample ID**  **Host** | **Host Species** | **Host COI** | **Expedition** | **Locality** | **Latitude** | **Longitude** | **COI** | **16S** | **H3** | **28S** | **ANT** |
| --- | --- | --- | --- | --- | --- | --- | --- | --- | --- | --- | --- | --- | --- |
| M13019a  M13019b  M13019c  M13019d  M13019e  M13019f  M13019g  M13020  M13023  M13024  M13026  M13030  M13031  M13033a  M13033b  M13039a  M13039b  M13039c  M13039d  P00472  P00408  M13035  M13037  M13018  M13021a  M13021b  M13021c  M13021d  M13021e  M13022  M13042a  M13042b  M12882a  M12882b  M12928  M13618  M13038a  M13038b  M13046  M13047a  M13047b  M13048a  M13048b  M13025  M13032  M13034  M13036  M13040  M13041  M13044  M13045  M13054a  M13054b  M13054c  M13054d  P00388  P00473  M13027  M13028  M13043a  M13043b | *A. perknasteri*  *A. perknasteri*  *A. perknasteri*  *A. perknasteri*  *A. perknasteri*  *A. perknasteri*  *A. perknasteri*  *A. perknasteri*  *A. perknasteri*  *A. perknasteri*  *A. perknasteri*  *A. perknasteri*  *A. perknasteri*  *A. perknasteri*  *A. perknasteri*  *A. perknasteri*  *A. perknasteri*  *A. perknasteri*  *A. perknasteri*  *A. perknasteri*  *A.* sp. 1  *A.* sp. 2  *A.* sp. 2  *A.* sp. 3  *A.* sp. 3  *A.* sp. 3  *A.* sp. 3  *A.* sp. 3  *A.* sp. 3  *A.* sp. 3  *A.* sp. 4  *A.* sp. 4  *A.* sp. 5  *A.* sp. 5  *A.* sp. 5  *A.* sp. 5  *A.* sp. 6  *A.* sp. 6  *A.* sp. 6  *A.* sp. 6  *A.* sp. 6  *A.* sp. 6  *A.* sp. 6  *A.* sp. 7  *A.* sp. 7  *A.* sp. 7  *A.* sp. 7  *A.* sp. 7  *A.* sp. 7  *A.* sp. 7  *A.* sp. 7  *A.* sp. 7  *A.* sp. 7  *A.* sp. 7  *A.* sp. 7  *A.* sp. 7  *A.* sp. 7  *A.* sp. 8  *A.* sp. 8  *A.* sp. 8  *A.* sp. 8 | P00384  P00384  P00384  P00384  P00384  P00384  P00384  P00364  P00373  P00365  P00371  P00423 P00407  P00363  P00363  P00406  P00406  P00406  P00406  P00472  P00408  P00420  P00419  P00409  P00376  P00376  P00376  P00376  P00376  P00029  P00527  P00527  S3631  S3631  E5052  E7111  P00361  P00361  P00347  P00362  P00362  P00354  P00354  P00418  P00003  E7112  P00526  P00348  P00350  P00528  P00393  P00355  P00355  P00355  P00355  P00388  P00473  P00422  P00417  P00387  P00387 | *Perknaster* cf. *densus*  *Perknaster* cf. *densus*  *Perknaster* cf. *densus*  *Perknaster* cf. *densus*  *Perknaster* cf. *densus*  *Perknaster* cf. *densus*  *Perknaster* cf. *densus*  *Perknaster* cf. *densus*  *Perknaster* cf. *densus*  *Perknaster* cf. *densus*  *Perknaster **  *Perknaster* sp. 1  *Perknaster aurorae*  *Perknaster* cf. *densus*  *Perknaster* cf. *densus*  *Perknaster antarcticus*  *Perknaster antarcticus*  *Perknaster antarcticus*  *Perknaster antarcticus*  *Perknaster* cf. *densus*  *Psilaster charcoti*  *Pteraster* sp. 1  *Pteraster* sp. 1  *Rhopiella hirsuta*  *Rhopiella* sp. 1  *Rhopiella* sp. 1  *Rhopiella* sp. 1  *Rhopiella* sp. 1  *Rhopiella* sp. 1  *Rhopiella hirsuta*  *Diplasterias* sp. 1  *Diplasterias* sp. 1  *Labidiaster annulatus**  *Labidiaster annulatus**  *Labidiaster annulatus**  *Labidiaster annulatus*  *Lysasterias heteractis*  *Lysasterias heteractis*  *Lysasterias perrieri*  *Lysasterias heteractis*  *Lysasterias heteractis*  *Lysasterias perrieri*  *Lysasterias perrieri*  *Notasterias* sp. 1  *Notasterias* sp. 1  *Notasterias* sp. 1  *Notasterias* sp. 1  *Notasterias* sp. 1  *Notasterias* sp. 1  *Notasterias* sp. 1  *Notasterias* sp. 1  *Notasterias* sp. 1  *Notasterias* sp. 1  *Notasterias* sp. 1  *Notasterias* sp. 1  *Notasterias* sp. 1  *Notasterias* sp. 1  *Paralophaster antarcticus*  *Lophaster gaini*  *Lophaster gaini*  *Lophaster gaini* | MN249412  MN249412  MN249412  MN249412  MN249412  MN249412  MN249412  MN249413  MN249419  MN249431  MN249442  MN249414  MN249416  MN249416  MN249423  MN249423  MN249423  MN249423  MN249428  MN249426  MN249437  MN249435  MN249411  MN249410  MN249410  MN249410  MN249410  MN249410  MN249432  MN249430  MN249430  MN249443  MN249420  MN249420  MN249433  MN249434  MN249434  MN249439  MN249439  MN249415  MN249441  MN249444  MN249417  MN249429  MN249421  MN249438  MN249427  MN249424  MN249424  MN249424  MN249424  MN249425  MN249440  MN249418  MN249436  MN249422  MN249422 | Polarstern 2012  Polarstern 2012  Polarstern 2012  Polarstern 2012  Polarstern 2012  Polarstern 2012  Polarstern 2012  Polarstern 2012  Polarstern 2012  Polarstern 2012  Polarstern 2012  Polarstern 2012  Polarstern 2012  Polarstern 2012  Polarstern 2012  Polarstern 2012  Polarstern 2012  Polarstern 2012  Polarstern 2012  Polarstern 2012  Polarstern 2012  Polarstern 2012  Polarstern 2012  Polarstern 2012  Polarstern 2012  Polarstern 2012  Polarstern 2012  Polarstern 2012  Polarstern 2012  Polarstern 2012  Polarstern 2012  Polarstern 2012  NB Palmer 2011  NB Palmer 2011  NB Palmer 2011  NB Palmer 2013  Polarstern 2012  Polarstern 2012  Polarstern 2012  Polarstern 2012  Polarstern 2012  Polarstern 2012  Polarstern 2012  Polarstern 2012  Polarstern 2012  Polarstern 2012  Polarstern 2012  Polarstern 2012  Polarstern 2012  Polarstern 2012  Polarstern 2012  Polarstern 2012  Polarstern 2012  Polarstern 2012  Polarstern 2012  Polarstern 2012  Polarstern 2012  Polarstern 2012  Polarstern 2012  Polarstern 2012  Polarstern 2012 | South Shetland Islands  South Shetland Islands  South Shetland Islands  South Shetland Islands  South Shetland Islands  South Shetland Islands  South Shetland Islands  Elephant Island  Elephant Island  Elephant Island  Elephant Island  South Shetland Islands  South Shetland Islands  Elephant Island  Elephant Island  South Shetland Islands  South Shetland Islands  South Shetland Islands  South Shetland Islands  South Shetland Islands  South Shetland Islands  South Shetland Islands  South Shetland Islands  South Shetland Islands  South Shetland Islands  South Shetland Islands  South Shetland Islands  South Shetland Islands  South Shetland Islands  South Shetland Islands  Elephant Island  Elephant Island  Shag Rocks  Shag Rocks  Shag Rocks  South Georgia  Elephant Island  Elephant Island  Elephant Island  Elephant Island  Elephant Island  Elephant Island  Elephant Island  South Shetland Islands  Elephant Island  Elephant Island  Elephant Island  Elephant Island  Elephant Island  Elephant Island  South Shetland Islands  Elephant Island  Elephant Island  Elephant Island  Elephant Island  South Shetland Islands  South Shetland Islands  Elephant Island  South Shetland Islands  South Shetland Islands  South Shetland Islands | -61.598  -61.598  -61.598  -61.598  -61.598  -61.598  -61.598  -61.2903  -61.2903  -61.2903  -61.2903  -61.8005  -61.8623  -61.2903  -61.2903  -61.9998  -61.9998  -61.9998  -61.9998  -62.5833  -61.8623  -61.8005  -61.8005  -61.8623  -61.825  -61.825  -61.825  -61.825  -61.825  -61.3457  -61.0292  -61.0292  -53.5171  -53.5171  -53.7211  -53.7151  -61.2903  -61.2903  -61.0181  -61.2903  -61.2903  -61.0113  -61.0113  -61.7638  -60.8693  -61.0292  -61.0292  -61.0181  -61.0181  -61.0292  -62.3698  -61.0113  -61.0113  -61.0113  -61.0113  -61.6477  -62.5833  -61.2903  -61.7638  -61.7533  -61.7533 | -57.278  -57.278  -57.278  -57.278  -57.278  -57.278  -57.278  -56.0208  -56.0208  -56.0208  -56.0208  -58.4873  -59.2578  -56.0208  -56.0208  -59.2455  -59.2455  -59.2455  -59.2455  -59.9166  -59.2578  -58.4873  -58.4873  -59.2578  -57.3935  -57.3935  -57.3935  -57.3935  -57.3935  -55.1833  -55.8083  -55.8083  -41.6288  -41.6288  -41.4627  -36.8357  -56.0208  -56.0208  -55.8448  -56.0208  -56.0208  -55.9672  -55.9672  -58.5015  -55.5025  -55.8083  -55.8083  -55.8448  -55.8448  -55.8083  -61.4296  -55.9672  -55.9672  -55.9672  -55.9672  -57.792  -59.9166  -56.0208  -58.5015  -58.5248  -58.5248 | MN224320  MN224321  MN224315  MN224316  MN224317  MN224318  MN224322  MN224323  MN224330  MN224331  MN224306  MN224334  MN224335  MN224310  MN224337  MN224342  MN224312  MN224343  MN224344  MN224362  MN224361  MN224338  MN224340  MN224319  MN224324  MN224325  MN224326  MN224327  MN224328  MN224329  MN224347  MN224348  MN224308  MN224341  MN224311  MN224353  MN224313  MN224354  MN224314  MN224355  MN224309  MN224336  MN224307  MN224339  MN224345  MN224346  MN224351  MN224352  MN224356  MN224357  MN224358  MN224359  MN224360  MN224363  MN224332  MN224333  MN224349  MN224350 | MN224437  MN224438  MN224439  MN224445  MN224446  MN224427  MN224447  MN224448  MN224450  MN224451  MN224454  MN224455  MN224456  MN224457  MN224433  MN224436  MN224440  MN224441  MN224442  MN224443  MN224444  MN224460  MN224431  MN224430  MN224428  MN224429  MN224452  MN224453  MN224464  MN224465  MN224466  MN224467  MN224434  MN224449  MN224458  MN224459  MN224463  MN224435  MN224432  MN224461  MN224462 | MN224387  MN224388  MN224389  MN224390  MN224397  MN224398  MN224364  MN224400  MN224401  MN224403  MN224404  MN224410  MN224411  MN224412  MN224369  MN224405  MN224407  MN224386  MN224391  MN224392  MN224393  MN224394  MN224395  MN224396  MN224372  MN224367  MN224366  MN224368  MN224408  MN224409  MN224377  MN224378  MN224379  MN224380  MN224381  MN224402  MN224365  MN224406  MN224370  MN224371  MN224375  MN224376  MN224382  MN224383  MN224384  MN224385  MN224399  MN224373  MN224374 | MN224470  MN224471  MN224472  MN224473  MN224480  MN224481  MN224468  MN224483  MN224484  MN224486  MN224487  MN224492  MN224493  MN224494  MN224495  MN224488  MN224489  MN224469  MN224474  MN224475  MN224476  MN224477  MN224478  MN224479  MN224497  MN224490  MN224491  MN224501  MN224502  MN224503  MN224504  MN224485  MN224496  MN224500  MN224505  MN224482  MN224498  MN224499 | MN224420  MN224423  MN224418  MN224422  MN224421  MN224413  MN224415  MN224419  MN224417  MN224416  MN224425  MN224424  MN224426 |
